# Hospital readmission among people experiencing homelessness in England: a cohort study of 2772 matched homeless and housed inpatients

**DOI:** 10.1136/jech-2020-215204

**Published:** 2021-01-05

**Authors:** Dan Lewer, Dee Menezes, Michelle Cornes, Ruth M Blackburn, Richard Byng, Michael Clark, Spiros Denaxas, Hannah Evans, James Fuller, Nigel Hewett, Alan Kilmister, Serena April Luchenski, Jill Manthorpe, Martin McKee, Joanne Neale, Alistair Story, Michela Tinelli, Martin Whiteford, Fatima Wurie, Alexei Yavlinsky, Andrew Hayward, Robert Aldridge

**Affiliations:** 1 Institute of Health Informatics, University College London, London, UK; 2 Collaborative Centre for Inclusion Health, University College London, London, UK; 3 Institute of Epidemiology and Health Care, University College London, London, UK; 4 NIHR Policy Research Unit in Health and Social Care Workforce, King's College London, London, UK; 5 Community and Primary Care Research Group, University of Plymouth, Plymouth, UK; 6 Care Policy and Evaluation Centre, The London School of Economics and Political Science, London, UK; 7 Alan Turing Institute, British Library, London, UK; 8 Pathway Charity, London, UK; 9 Department of Health Services Research and Policy, London School of Hygiene & Tropical Medicine, London, UK; 10 National Addiction Centre, Institute of Psychiatry, Psychology & Neuroscience, King's College London, London, UK; 11 Find & Treat, University College London Hospitals NHS Foundation Trust, London, UK; 12 Department of Nursing & Community Health, Glasgow Caledonian University, Glasgow, UK

**Keywords:** homelessness, access to hlth care, health inequalities, record linkage

## Abstract

**Background:**

Inpatients experiencing homelessness are often discharged to unstable accommodation or the street, which may increase the risk of readmission.

**Methods:**

We conducted a cohort study of 2772 homeless patients discharged after an emergency admission at 78 hospitals across England between November 2013 and November 2016. For each individual, we selected a housed patient who lived in a socioeconomically deprived area, matched on age, sex, hospital, and year of discharge. Counts of emergency readmissions, planned readmissions, and Accident and Emergency (A&E) visits post-discharge were derived from national hospital databases, with a median of 2.8 years of follow-up. We estimated the cumulative incidence of readmission over 12 months, and used negative binomial regression to estimate rate ratios.

**Results:**

After adjusting for health measured at the index admission, homeless patients had 2.49 (95% CI 2.29 to 2.70) times the rate of emergency readmission, 0.60 (95% CI 0.53 to 0.68) times the rate of planned readmission and 2.57 (95% CI 2.41 to 2.73) times the rate of A&E visits compared with housed patients. The 12-month risk of emergency readmission was higher for homeless patients (61%, 95% CI 59% to 64%) than housed patients (33%, 95% CI 30% to 36%); and the risk of planned readmission was lower for homeless patients (17%, 95% CI 14% to 19%) than for housed patients (30%, 95% CI 28% to 32%). While the risk of emergency readmission varied with the reason for admission for housed patients, for example being higher for admissions due to cancers than for those due to accidents, the risk was high across all causes for homeless patients.

**Conclusions:**

Hospital patients experiencing homelessness have high rates of emergency readmission that are not explained by health. This highlights the need for discharge arrangements that address their health, housing and social care needs.

## Introduction

Homelessness is an enduring social problem that is associated with poor health outcomes, with cohort studies showing mortality rates of three to six times the general population.^1–4^ Although the size and structure of the homeless population are difficult to estimate, data in England suggest steep increases in recent years. The number of people sleeping rough identified by official counts increased from 1353 in 2010 to 4266 in 2019,^5^ with actual numbers likely to be greater than this. The same period also saw a steep increase in hospital attendances for people with ‘no fixed abode’.^6^

Leaving hospital is often a traumatic experience for people without a fixed address, and surveys suggest that 30%–70% of homeless inpatients are ‘discharged to the street’ (ie, sleeping rough immediately after discharge).^7 8^ Recovery after a hospital admission may be spent sleeping rough or in insecure accommodation such as hostels or sofa-surfing. In addition, access to ongoing community healthcare may be poor. Qualitative research has identified barriers including stigmatisation when accessing health services, primary healthcare practitioners refusing to register homeless people, and priorities that compete with health such as arranging accommodation.^9–11^ As a result, outcomes after hospital discharge may be poor, and studies in the USA have shown that homeless inpatients are more likely to be readmitted than housed inpatients.^12–15^

In response to concerns about poor discharge arrangements for homeless inpatients, the UK government set up the ‘Homeless Hospital Discharge Fund’,^16^ which funded partnerships of National Health Service (NHS) and non-profit organisations to develop methods of supported discharge. These schemes operated between 2013 and 2016 and used a range of models. Most included a housing specialist who helped patients access community health and housing services. In some schemes, discharge was managed by a multidisciplinary team including general practitioners (GPs), nurses, therapists, and housing workers. Some had accompanying intermediate care facilities providing accommodation and clinical support. Data collected by the schemes give a detailed insight into the outcomes of homeless inpatients after discharge from hospital.

Our previous analysis of linked death records for people attending the homeless hospital discharge schemes found that deaths after discharge were related to a wide range of physical health problems, with alcohol, drugs or suicide (sometimes considered the main health problems in this population) the primary cause for only one-third of deaths.^17^ In this present analysis we compare the risk of hospital readmission among homeless inpatients with housed inpatients living in socioeconomically deprived areas.

## Methods

We conducted a retrospective cohort study of the rate of hospital readmission after an emergency hospital admission, comparing homeless patients with housed patients living in socioeconomically deprived areas.

### Data source

The data were collected as part of an evaluation of hospital discharge schemes established through the Homeless Hospital Discharge Fund.^16^ We worked with 17 schemes covering 78 hospitals across England between November 2013 and November 2016. Schemes did not adopt a common definition of homelessness and had various ways of identifying eligible patients. Some relied on referrals from clinicians and others did ward rounds. Services primarily focused on people living on the streets or in night shelters and hostels for single homeless people, but may also have worked with people who meet broader definitions of homelessness such as people who are sofa-surfing, or at risk of losing an existing tenancy. Teams helped plan discharge and helped patients access housing, intermediate care, and other services that were available locally. Full details of the datasets are available in a prepublished protocol.^18^

### Data cleaning and linkage

We collected patient identifiers from the 17 homeless discharge schemes. We excluded patients under the age of 18 years and those with insufficient data for record linkage. We sent the identifiers to NHS Digital for deterministic linkage^19^ with national hospital and mortality databases (‘Hospital Episode Statistics’ and the Office for National Statistics mortality database respectively). Linked data were available until 31 March 2018. We considered the ‘index’ admission as the first time a homeless patient was seen by a hospital discharge service. The admission dates provided by the homeless discharge schemes sometimes varied from those recorded in the national hospital database, and we used an algorithm to select the index admission (see online supplemental information). For comparison purposes, we requested data on hospital episodes and deaths for a sample of housed inpatients admitted to the same 78 hospitals between November 2013 and November 2016, with a home address in the most deprived quintile of neighbourhoods based on the Index of Multiple Deprivation,^20^ and who were not seen by a homeless discharge scheme. We processed the hospital episode data into ‘spells’ (or ‘admissions’), because English hospital data are structured such that a single hospital spell is sometimes divided into several episodes of care led by different doctors or departments. A spell represents a continuous period of time in hospital.

10.1136/jech-2020-215204.supp1 Supplementary data

### Matching

**Figure 1:**
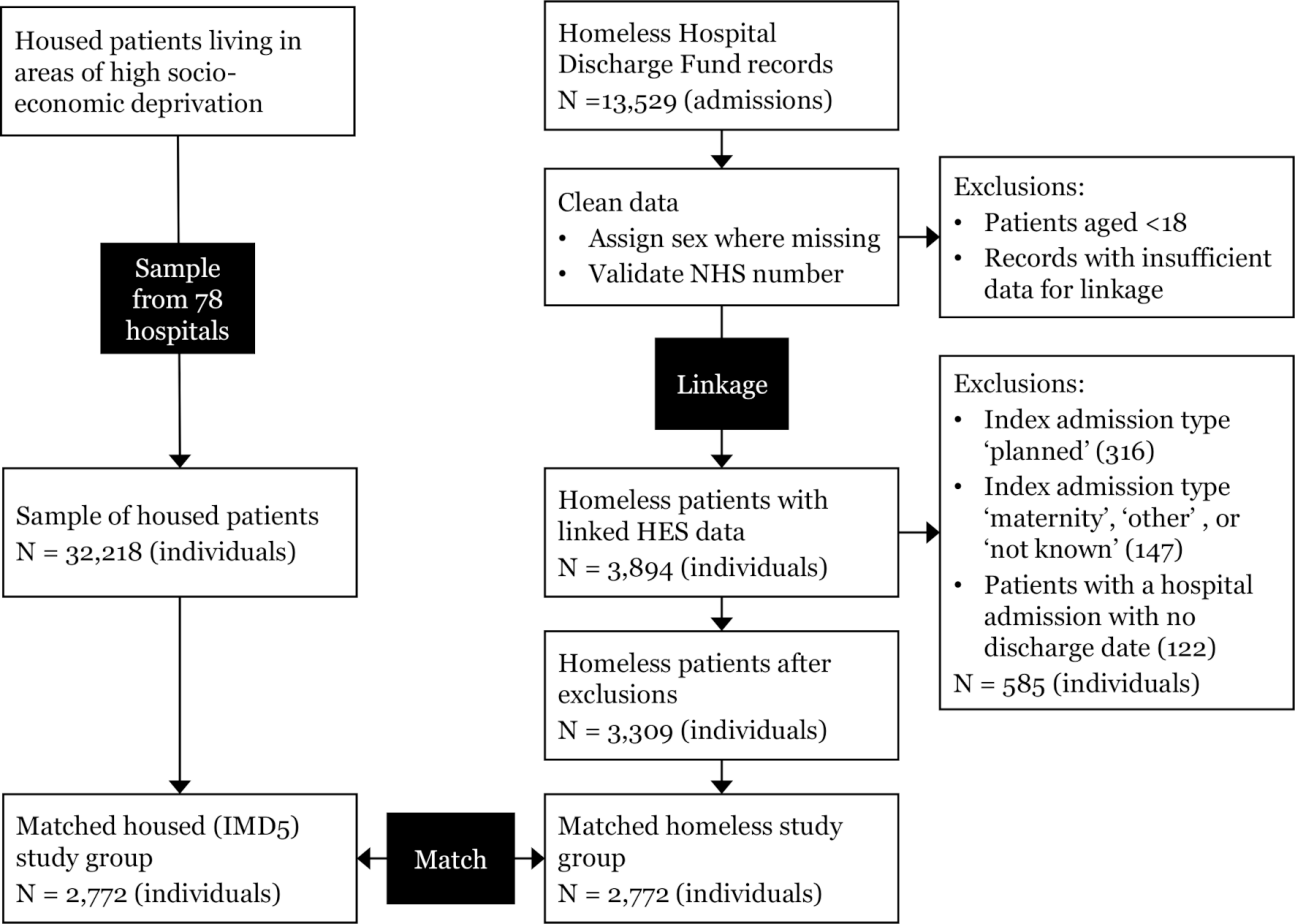
Derivation of the study cohort. HES, Hospital Episode Statistics; NHS. National Health Service.

For this analysis, we only included patients who were admitted in an emergency. Planned admissions were rare in the homeless group (8% of all admissions) and often represent healthcare that can be conducted on a single day, such as dialysis or physical rehabilitation, and readmission may not be related to poor discharge arrangements or ongoing care. We used a matched design to compare homeless and housed patients. For each homeless patient we selected at random one housed patient of the same sex and age group (using age groups 18–24, 25–34 and then 10-year age groups) who was discharged alive from the same hospital in the same year. We have published our matching algorithm at https://github.com/danlewer/homeless-discharge/. We used this design to allow definition of an ‘index’ admission for housed patients, while avoiding potential biases in choosing an index date that may result from other approaches (such as selecting an admission at random, which is likely to select patients during periods of poor health). The derivation of the study cohort is shown in figure 1.

### Outcomes

Outcomes were the counts of planned hospital readmissions, emergency hospital readmissions, and Accident and Emergency (A&E) visits, as defined in the study protocol.^18^

### Covariates

We defined the number of comorbidities as the number of ICD-10 (10th revision of the International Statistical Classification of Diseases and Related Health Problems) chapters that were present in the primary diagnosis field for hospital admissions in the 4 years prior to the index date. We used 4 years because all participants had at least 4 years of prior data). We only counted comorbidities from ICD-10 chapters 2–14 and 17,^21^ excluding chapters such as infections where an admission may not represent a long-term condition. We defined the reason for index admission using the ICD-10 chapter of the primary diagnosis, grouping chapters accounting for fewer than 100 index admissions as ‘other’ (see table 1 for a list of ICD-10 chapters included in the analysis). For descriptive purposes, we also reported whether discharge from the index admission was against medical advice, and whether patients died during follow-up.

**Table 1:** Characteristics of hospital patient experiencing homelessness, compared with housed patients living in socioeconomically deprived areas

Variable	Level	Homeless, n (%)	Housed, n (%)
Total		2772 (100)	2772 (100)
Age at index admission (matched)	Mean (SD)	44.21 (14.15)	44.34 (14.56)
	Median (IQR)	43.64 (33.37–53.75)	43.67 (33.05–53.51)
Sex (matched)	Female	768 (28)	768 (28)
	Male	2004 (72)	2004 (72)
Year of index admission (matched)	2013	76 (3)	76 (3)
	2014	769 (28)	769 (28)
	2015	948 (34)	948 (34)
	2016	979 (35)	979 (35)
Number of comorbidities, based on prior hospital admissions (ICD-10 chapters 2–14 and 17)	0	926 (33)	1238 (45)
	1	769 (28)	784 (28)
	2	541 (20)	424 (15)
	3	307 (11)	183 (7)
	4+	229 (8)	143 (5)
	Mean (SD)	1.38 (1.41)	1.02 (1.24)
	Median (IQR)	1 (0–2)	1 (0–2)
ICD-10 chapter of index admission	Accidents and other external	695 (25)	452 (16)
	Digestive diseases	223 (8)	298 (11)
	Circulatory diseases	226 (8)	228 (8)
	Mental health	347 (13)	90 (3)
	Respiratory diseases	189 (7)	236 (9)
	Skin problems	206 (7)	106 (4)
	Genitourinary diseases	89 (3)	210 (8)
	Musculoskeletal problems	112 (4)	127 (5)
	Infections	75 (3)	78 (3)
	Cancers	63 (2)	80 (3)
	Other*	547 (20)	867 (31)
Discharge method for index admission	Self (without clinical consent)	253 (9)	100 (4)
	With clinical consent	2519 (91)	2672 (96)
Years of follow-up after index admission		6753	6987
Emergency readmissions (rate per 1000 person-years)		12 472 (1847)	4525 (648)
Planned readmissions (rate per 1000 person-years)		3400 (503)	4769 (683)
A&E visits (rate per 1000 person-years)		43 808 (6487)	14 186 (2030)
Died during the study (%)		451 (16)	311 (11)

*‘Other’ includes ICD-10 chapters (II) diseases of the blood and blood-forming organs; (IV) endocrine, nutritional and metabolic diseases; (VI) diseases of the nervous system; (VII) diseases of the eye; (VIII) diseases of the ear.

A&E, Accident and Emergency; ICD-10, 10th revision of the International Statistical Classification of Diseases and Related Health Problems.

### Statistical analysis

We estimated the probability of patients having at least one of each outcome (emergency readmission, planned readmission and A&E visit) in the 12 months after discharge, with each outcome stratified by the ICD-10 chapter of the index admission. We used the Kaplan-Meier method to estimate cumulative incidence at 12 months, because some participants had less than 12 months of follow-up, for example due to death. The SEs of the cumulative incidence were clustered by hospital to account for differences in clinical practice.

To estimate rate ratios comparing homeless and housed patients, we used a mixed negative binomial model for each outcome, with homeless/housed status as the main independent variable; adjustment for the matching variables age, sex, year of discharge and a random intercept for hospital site; and an offset for the log time-at-risk. We then additionally adjusted for the count of comorbidities and the reason for admission. We used negative binomial models because the outcomes were dispersed (ie, the variance of the count of readmissions was greater than the mean).

Analysis was conducted using R V.3.6.2.

## Results

A total of 3894 homeless patients were supported by the 17 specialist discharge services. Of these patients, 3309 were admitted in an emergency and eligible for inclusion. We matched 2772 (84%) of these patients to a unique housed patient. The remaining 537 could not be matched as there was no remaining eligible housed patient in our data. Unmatched homeless patients were slightly younger and more likely to be men than matched patients (online supplemental information).

Homeless patients had more comorbidities than housed patients and were more likely to be admitted for mental health problems or ‘external’ causes (including accidents). Table 1 shows the characteristics of homeless patients and matched housed patients.

**Figure 2:**
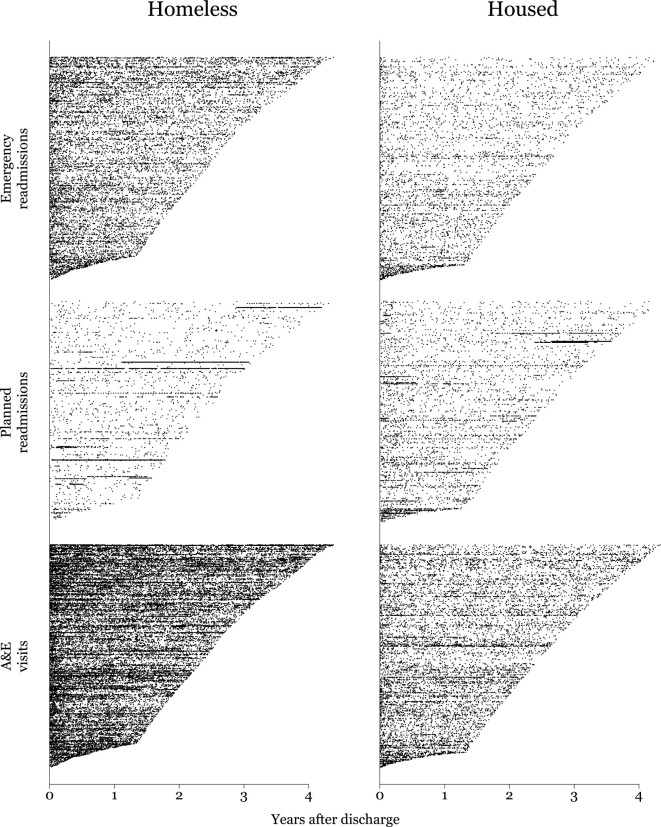
Plot of hospital spells and A&E visits after discharge, with patients arranged in rows and ordered by follow-up duration. Dots indicate readmission dates. Patients with no readmissions are represented as an empty (white) row. A&E, Accident and Emergency.

We plotted all readmissions and A&E visits for every patient in the study (figure 2). Visual inspection of this plot suggested that emergency readmissions and A&E visits occurred more frequently for patients experiencing homelessness at the index admission, while there was a similar rate of planned readmissions. There were more series of repeat planned admissions within the group experiencing homelessness (visible on the plot as solid horizontal lines). Consequently, we conducted an exploratory analysis where we defined a ‘series’ as weekly (or more frequent) planned admissions for at least 8 consecutive weeks. In the homeless group, there were 16 such series involving 9 patients. There were 1201 admissions within these series, of which 949/1201 (79%) had a primary diagnosis of renal failure and most had procedure codes for dialysis. This means that one-third (1201/3400; 35%) of the planned admissions in the homeless cohort related to these series. In the housed group, there were 11 series involving 7 patients. There were 571 admissions in these series and 174 (30%) had a primary diagnosis of renal failure. This means that one in eight (571/4769; 12%) of the planned admissions in the housed cohort related to these series.

**Figure 3:**
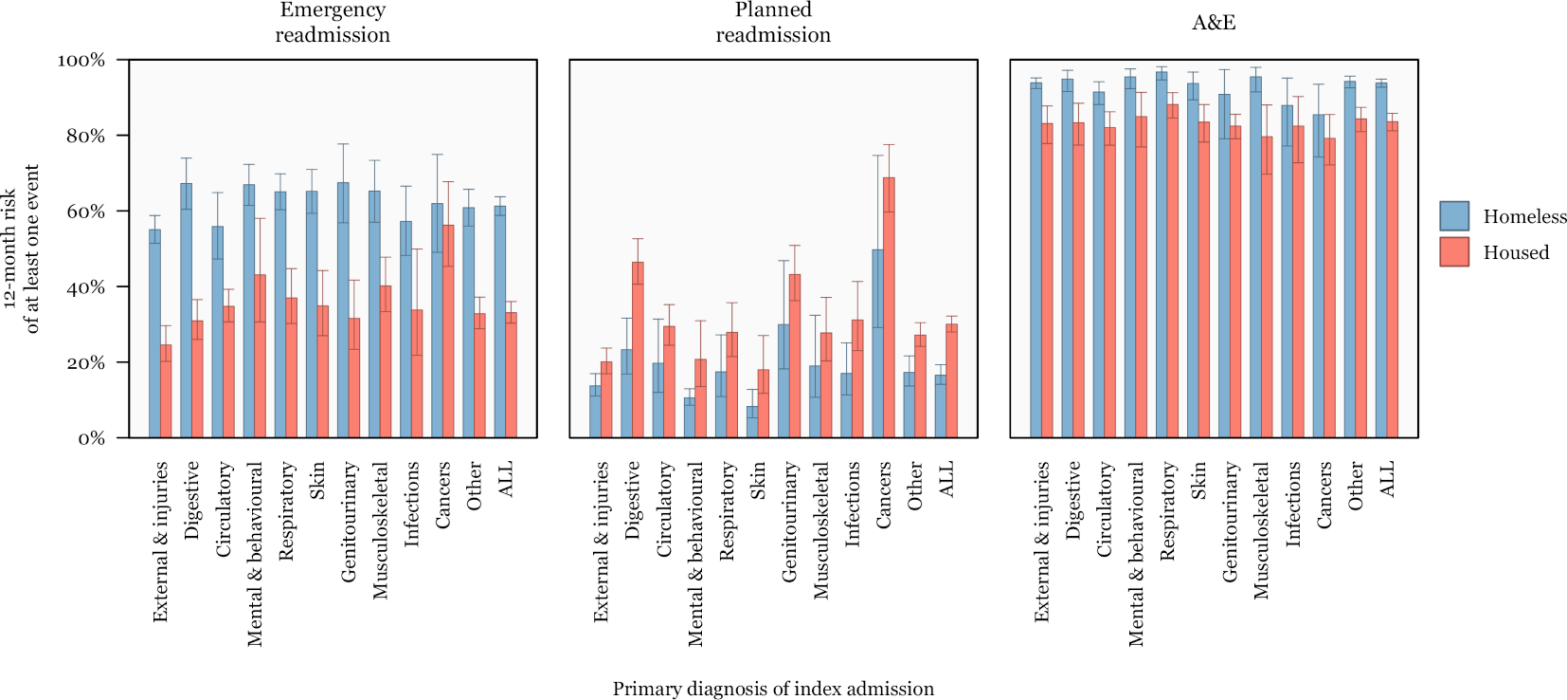
The 12-month risk of readmission, stratified by the ICD-10 chapter of index admission. A&E, Accident and Emergency; ICD-10, 10th revision of the International Statistical Classification of Diseases and Related Health Problems.

The 12-month risk of emergency readmission was 61% (95% CI 59% to 64%) for homeless patients and 33% (95% CI 30% to 36%) for housed patients; for planned readmission it was 17% (95% CI 14% to 19%) for homeless patients and 30% (95% CI 28% to 32%) for housed patients; and for A&E visits it was 94% (95% CI 93% to 95%) for homeless patients and 84% (95% CI 81% to 86%) for housed patients (figure 3). Among housed patients, the risk of emergency readmission varied substantially according to the cause of the index admission. For example, patients admitted with a primary cause of cancer had a 56% (95% CI 45% to 68%) risk of emergency readmission over the following 12 months, compared with 25% (95% CI 20% to 30%) for patients admitted following an accident. In contrast, the 12-month risk of an emergency readmission was greater than 50% regardless of the reason for the index admission, with limited variation.

After adjusting for comorbidities and the ICD-10 chapter of the index admission, patients experiencing homelessness had 2.49 (95% CI 2.29 to 2.70) times the rate of emergency readmissions, 0.60 (95% CI 0.53 to 0.68) times the rate of planned readmissions and 2.57 (95% CI 2.41 to 2.73) times the rate of A&E visits (table 2). As an exploratory sensitivity analysis (not prespecified), we fit the model for planned readmissions excluding ‘series’ of admissions (defined above), which gave a rate ratio of 0.51 (95% CI 0.45 to 0.57) when adjusting for matching variables only, and a fully adjusted ratio of 0.55 (95% CI 0.49 to 0.62), that is, a wider relative difference between homeless and housed patients after removing series of planned admissions.

**Table 2:** Incidence rate ratios of readmissions and A&E visits, comparing homeless patients with housed patients living in socioeconomically deprived areas (results of negative binomial regression)

	Adjusted on matching variables	Further adjusted for comorbidities and ICD-10 chapter of index admission
Emergency readmissions	2.92 (2.67–3.19) p<0.001	2.49 (2.29–2.70) p<0.001
Planned readmissions	0.63 (0.55–0.72) p<0.001	0.60 (0.53–0.68) p<0.001
A&E visits	3.06 (2.86–3.27) p<0.001	2.57 (2.41–2.73) p<0.001

A&E, Accident and Emergency; ICD-10, 10th revision of the International Statistical Classification of Diseases and Related Health Problems.

## Discussion

Hospital patients who are experiencing homelessness have high rates of emergency readmission and A&E visits after discharge. Following an acute illness, most patients are expected to recover in their own home, and the rate of readmission is relatively low. Our results show this is not the case for homeless patients.

Similar to our study, studies of hospital admissions among homeless populations in North America have reported diverse physical and mental health problems on admission and high rates of emergency readmission.^8 12–15^ Results are difficult to compare directly due to differences in the populations, healthcare systems, and study methodologies. A study in the USA found that 17% of inpatients with hospital records indicating homelessness were readmitted within 90 days; only slightly higher than 14% of housed patients.^15^ Another study, also using homelessness recorded in routine hospital data, found that homelessness was associated with 1.4 times the odds of readmission.^13^ These studies both found modest differences in readmission risk. Use of routinely captured data to identify homelessness may have led to inclusion of people experiencing less severe forms of homelessness, such as unstable or temporary housing. In contrast, two other studies in the USA measured hospital readmission for patients who live in homeless shelters, and are likely to have longer histories of homelessness and sleeping rough.^12 13^ These studies found that readmission risk was approximately three times that of housed patients; more similar to our estimates. It is also important to remember that the housed comparison group in our study live in areas of high socioeconomic deprivation. The difference in readmission risk between homeless individuals and the general population is likely to be wider, because socioeconomic deprivation is associated with morbidity and readmission.^22^

As far as we are aware, ours is the largest study of the outcomes of homeless patients after discharge from hospital in the UK. National data regarding inpatients with ‘no fixed abode’ show that the median age was 43, three-quarters were men and 9 out of 10 were admitted in an emergency.^23^ Our sample has similar characteristics, supporting generalisability between our results and homeless patients discharged from hospital nationally.

In our protocol^18^ we planned to analyse data from a ‘control’ group of homeless patients who were not seen by a specialist discharge scheme and were instead identified through linkage to an external service that supports people experiencing homelessness. This control group was intended to allow estimation of the effect of the specialist discharge schemes on readmission rates. However, we subsequently found problems with these data; most importantly that we could not confirm whether patients were homeless at the point of hospital admission (unlike those included in this analysis, whom specialist discharge teams identified as homeless). We therefore limited the present analysis to a comparison of readmission for homeless and housed patients. This means that our analysis primarily provides insight into readmissions for homeless people after hospital discharge, rather than an evaluation of the discharge schemes.

We did not account for competing risks in our analysis. Death is likely to be a competing risk for hospital readmission, on the assumption that patients who died would have an increased risk of readmission if they had survived. Homeless patients in our study had a higher risk of death (16% of homeless patients died, compared with 11% of housed patients) and the effect of competing risks is therefore likely to be that rate ratios are slightly understated. We did not account for competing risks to maximise the simplicity of the analysis, and because competing risks are unlikely to have an important bearing on the results.

We found that homeless inpatients had a lower rate of planned readmissions than housed inpatients. Our post-hoc visual inspection of the readmissions data (figure 2) suggested that the distribution of planned readmissions may be different in the two groups, with more ‘series’ of planned admissions for homeless patients. We found that these series related to a small number of individuals with large numbers of admissions, mainly for renal failure and dialysis. After removing these individuals from both groups, the difference between homeless and housed patients was wider. This low rate of planned care reflects barriers that have been observed in qualitative research,^9–11^ and suggests that long-term conditions are often managed in crises. The relatively large number of homeless patients with renal failure was not expected and is an avenue for further research. It may represent different patterns of healthcare use (for example dialysis being undertaken by housed patients in other settings), or a higher risk of renal failure related to use of drugs or alcohol, or cardiovascular disease. The importance of renal failure in terms of the quantity of healthcare would not have been identified from the index admission alone and reflects the strengths of using longitudinal data. Nephrologists in the USA have previously observed the difficulty of providing dialysis to homeless patients with end-stage renal disease.^24^

A range of intermediate care services has been developed in England for older people. These services are not easily accessible to homeless patients younger than 55 years who may also be frail or in need of rehabilitation or palliative care.^25 26^ The effectiveness of these services can be limited by shortages of longer term care, leading to intermediate care becoming ‘blocked’^27^ and contributing to bed shortages. In this context it is unsurprising that people experiencing homelessness, many of whom are middle-aged and do not meet eligibility criteria for services for older people, struggle to access existing intermediate care provision. Our results show a need for community ‘step-down’ services that provide ongoing care. Observational studies have found that step-down services are associated with reduced readmissions,^28–31^ and a trial of GP-led management of discharge in the UK found reduced street homelessness and improved quality of life.^32^

Hospital patients who are experiencing homelessness have extreme rates of emergency readmission after discharge, reflecting poor housing and ongoing community care that is designed around the needs of people who have stable housing. Ultimately, the inequality between housed and homeless people can only be addressed by preventing homelessness (Box 1).

Box 1 **Interpretation by an expert with lived experience**

For many people who are street homeless, hospital is an inhospitable, if not hostile environment. A single visit, or even street lore alone, can be enough to cause one to make inventive efforts to disguise one’s homelessness in order to receive less visceral and judgemental handling. Certainly, it is not an experience anyone would rush to embrace, hence the (potentially fatal) avoidance and delay before seeking treatment. Conversely, particularly for some of us with poor mental health, a hospital represents a building with ‘indoor’ comforts and facilities like heat, light and hot water and crucially, a place populated by people who are perceived to have a duty to play nicely. Perhaps this cohort of ‘regulars’ is partly responsible for the medical profession’s distaste of us as a whole. In any case, there are deep differences in how people who are homeless approach and are received by healthcare services, as opposed to those who are housed. This may explain the results under Discussion. If hospital Trusts were to adopt a less ‘gatekeeping’ approach to homeless patients, try not to refer to us as bed-blockers, at least not in our hearing, and provide timely treatments for our multiple morbidities, the costs of our care could be reduced dramatically. (JF)

**What is already known on this subject**

- Many people experiencing homelessness are discharged from hospital without secure accommodation. Rough sleeping and sofa-surfing are common during recuperation from an acute illness. Homelessness is also associated with poor access to community health and social care services. Studies in North America show that homelessness is associated with high risk of readmission.

**What this study adds**

- Hospital inpatients in England who are experiencing homelessness have a high risk of emergency readmission and Accident and Emergency visits after discharge, when compared with housed patients. Unlike housed patients, the risk of readmission among homeless inpatients is high regardless of the original cause of admission. Homeless inpatients are less likely to have planned readmissions, which may reflect poor ongoing care. Hospitals need specialist discharge schemes that work alongside community support services, including housing and social care.

## Data Availability

Data may be obtained from a third party and are not publicly available. As part of the approvals and information governance frameworks, we are unable to share the underlying data for this research study. Our approval only allowed researchers involved in this specific project to access data for the prespecified and approved analyses. Therefore, data collection and linkage would have to be repeated with new approvals sought by anyone wanting access to the underlying data used in this analysis. Application for access should be directed to the CAG of the Health Research Authority. Information regarding the application process and relevant links for applications are available from the CAG website.
