# Supplementary material for: Hospital readmission among people experiencing homelessness in England: a cohort study of 2772 matched homeless and housed inpatients
Source: J Epidemiol Community Health. 2021 Jan 5;75(7):681–8. doi: 10.1136/jech-2020-215204 (PMC8223662; doi:10.1136/jech-2020-215204)

BMJ Publishing Group Limited (BMJ) disclaims all liability and responsibility arising from any reliance placed on this supplemental material which has been supplied by the author(s)

Supplementary Information

1 Comparison of matched and unmatched homeless participants 2
2 Full results of regression models comparing homeless and housed participants 3
3 Cumulative incidence of readmission at 12 months, stratified by reason for index admission 5
4 Algorithm for selecting index admission of homeless participants 6

Lewer D, *et al. J Epidemiol Community Health* 2021; 75:681–688. doi: 10.1136/jech-2020-215204

1 Comparison of matched and unmatched homeless participants

Variable	Level	Matched n (%)	Unmatched n (%)
Total		2,772 (100)	537 (100)
Age	Mean (sd)	44.21 (14.15)	41.14 (11.85)
	Median (IQR)	43.64 (33.37-53.75)	40.92 (32.97-48.94)
Sex	Female	768 (28)	80 (15)
	Male	2,004 (72)	457 (85)
Year of index	2013	76 (3)	30 (6)
	2014	769 (28)	130 (24)
	2015	948 (34)	155 (29)
	2016	979 (35)	222 (41)
Comorbidities	0	926 (33)	183 (34)
	1	769 (28)	170 (32)
	2	541 (20)	94 (18)
	3	307 (11)	53 (10)
	4+	229 (8)	37 (7)
	Mean (sd)	1.38 (1.41)	1.28 (1.35)
	Median (IQR)	1 (0-2)	1 (0-2)
Reason for index admission	External	695 (25)	131 (24)
	Digestive	223 (8)	28 (5)
	Circulatory	226 (8)	25 (5)
	Mental health	347 (13)	156 (29)
	Respiratory	189 (7)	19 (4)
	Skin	206 (7)	29 (5)
	Genitourinary	89 (3)	9 (2)
	Musculoskeletal	112 (4)	17 (3)
	Infections	75 (3)	10 (2)
	Cancers	63 (2)	6 (1)
	Other	547 (20)	107 (20)
Type of discharge[a]	Normal	2,519 (91)	487 (91)
	DAMA	253 (9)	50 (9)

[a] Normal = With medical consent. DAMA = Discharge against medical advice.

Lewer D, *et al. J Epidemiol Community Health* 2021; 75:681–688. doi: 10.1136/jech-2020-215204

2 Full results of regression models comparing homeless and housed participants

We used mixed negative binomial models with the count of readmissions as the dependent variable and the homeless or housed status of the patient as the main independent variable. Other independent variables are shown in the table below, plus an offset for the log follow-up time. In adjusted models, a random effect is included for the hospital site. The values in the table are Incident Rate Ratios with 95% confidence intervals.

Variable	Level	Unadjusted	Adjusted for matching variables[b]	Fully adjusted
**Emergency readmissions**				
Group	Housed (ref)	1	1	1
	Homeless	2.69 (2.46, 2.94)	2.92 (2.67, 3.19)	2.49 (2.29, 2.70)
Sex	Female (ref)	1		
	Male	0.93 (0.84, 1.03)		
Age		1.16 (1.10, 1.21)		
Comorbidities	0 (ref)	1	1	
	1	2.76 (2.49, 3.06)	2.73 (2.46, 3.03)	
	2	4.86 (4.32, 5.47)	4.78 (4.24, 5.38)	
	3	6.37 (5.50, 7.38)	6.17 (5.32, 7.16)	
	4+	12.65 (10.78, 14.86)	12.37 (10.52, 14.56)	
Reason for index admission	External (ref)	1	1	
	Cancers	1.72 (1.24, 2.38)	1.52 (1.09, 2.11)	
	Circulatory	1.02 (0.84, 1.24)	0.91 (0.75, 1.11)	
	Digestive	1.22 (1.02, 1.47)	1.17 (0.98, 1.41)	
	Genitourinary	1.08 (0.86, 1.35)	0.95 (0.76, 1.19)	
	Infections	1.05 (0.78, 1.41)	1.00 (0.75, 1.35)	
	Musculoskeletal	1.18 (0.93, 1.51)	1.11 (0.87, 1.41)	
	Mental health	1.82 (1.51, 2.20)	1.75 (1.45, 2.11)	
	Other	1.19 (1.03, 1.36)	1.13 (0.98, 1.29)	
	Respiratory	1.67 (1.38, 2.03)	1.45 (1.19, 1.76)	
	Skin	1.02 (0.82, 1.27)	0.97 (0.78, 1.20)	
Year of index admission	2013 (ref)	1		
	2014	0.76 (0.57, 1.01)		
	2015	0.62 (0.47, 0.82)		
	2016	0.76 (0.57, 1.00)		
**Planned readmissions**				
Group	Housed (ref)	1	1	1
	Homeless	0.62 (0.54, 0.70)	0.63 (0.55, 0.72)	0.60 (0.53, 0.68)
Sex	Female (ref)	1		
	Male	0.90 (0.78, 1.04)		
Age		1.35 (1.26, 1.44)		
Comorbidities	0 (ref)	1	1	
	1	1.93 (1.64, 2.26)	1.87 (1.60, 2.19)	
	2	2.49 (2.06, 2.99)	2.37 (1.97, 2.85)	
	3	3.62 (2.87, 4.55)	3.43 (2.72, 4.32)	
	4+	8.65 (6.72, 11.12)	8.09 (6.29, 10.42)	

[b] Homeless and housed groups were matched on age group, sex, year of discharge, and hospital site. Hospital site is included as a random effect. Age is standardised such that the coefficient represents the association with one standard deviation in age.

Variable	Level	Unadjusted	Adjusted for matching variables[b]	Fully adjusted
Reason for index admission	External (ref)	1	1	
	Cancers	15.41 (10.28, 23.12)	15.21 (10.20, 22.69)	
	Circulatory	1.65 (1.26, 2.16)	1.26 (0.96, 1.66)	
	Digestive	2.12 (1.65, 2.74)	2.04 (1.58, 2.63)	
	Genitourinary	4.35 (3.23, 5.85)	3.50 (2.61, 4.71)	
	Infections	2.85 (1.92, 4.23)	2.96 (2.01, 4.36)	
	Musculoskeletal	1.76 (1.25, 2.46)	1.43 (1.02, 2.00)	
	Mental health	0.75 (0.57, 1.01)	0.74 (0.56, 0.99)	
	Other	1.97 (1.63, 2.39)	1.92 (1.58, 2.32)	
	Respiratory	3.24 (2.49, 4.23)	2.90 (2.23, 3.78)	
	Skin	1.97 (1.47, 2.66)	1.80 (1.33, 2.42)	
Year of index admission	2013 (ref)	1		
	2014	0.79 (0.53, 1.19)		
	2015	0.95 (0.63, 1.42)		
	2016	1.11 (0.74, 1.66)		
**A&E visits**				
Group	Housed (ref)	1	1	1
	Homeless	3.02 (2.82, 3.22)	3.06 (2.86, 3.27)	2.57 (2.41, 2.73)
Sex	Female (ref)	1		
	Male	1.01 (0.93, 1.09)		
Age		0.99 (0.96, 1.03)		
Comorbidities	0 (ref)	1	1	
	1	2.19 (2.03, 2.37)	2.24 (2.07, 2.43)	
	2	3.26 (2.97, 3.58)	3.32 (3.03, 3.65)	
	3	4.13 (3.67, 4.64)	4.39 (3.90, 4.94)	
	4+	8.16 (7.16, 9.30)	8.65 (7.58, 9.86)	
Reason for index admission	External (ref)	1	1	
	Cancers	0.78 (0.61, 1.01)	0.77 (0.60, 0.99)	
	Circulatory	0.67 (0.58, 0.78)	0.67 (0.58, 0.78)	
	Digestive	1.06 (0.92, 1.21)	1.05 (0.91, 1.20)	
	Genitourinary	0.80 (0.67, 0.94)	0.76 (0.64, 0.90)	
	Infections	0.82 (0.66, 1.03)	0.83 (0.66, 1.03)	
	Musculoskeletal	1.19 (0.99, 1.43)	1.20 (1.00, 1.44)	
	Mental health	1.94 (1.68, 2.25)	1.92 (1.66, 2.22)	
	Other	1.04 (0.94, 1.16)	1.04 (0.94, 1.15)	
	Respiratory	1.36 (1.17, 1.57)	1.33 (1.15, 1.54)	
	Skin	0.92 (0.78, 1.08)	0.91 (0.77, 1.07)	
Year of index admission	2013 (ref)	1		
	2014	0.65 (0.52, 0.80)		
	2015	0.59 (0.48, 0.73)		
	2016	0.68 (0.55, 0.85)		

3 Cumulative incidence of readmission at 12 months, stratified by reason for index admission

We used the Kaplan-Meier method to estimate the cumulative incidence of readmission during the first 12 months after discharge (i.e. the risk of one or more readmissions), with censoring at death or end-of-follow-up. The values in the table below are percentages with 95% confidence intervals.

**ICD-10 chapter of index admission**	**Homeless**	**Housed**
Emergency readmissions		
Cancers	61.9 (49.0-74.9)	56.2 (45.3-67.7)
Circulatory	55.9 (47.3-64.8)	34.8 (30.6-39.2)
Digestive	67.3 (60.4-74.0)	30.9 (26.0-36.5)
External & injuries	55.0 (51.4-58.8)	24.5 (20.2-29.7)
Genitourinary	67.5 (56.8-77.7)	31.6 (23.4-41.7)
Infections	57.2 (48.2-66.5)	33.8 (21.8-49.9)
Mental & behavioural	66.9 (61.4-72.3)	43.1 (30.6-58.0)
Musculoskeletal	65.2 (57.0-73.4)	40.1 (33.3-47.8)
Other	60.8 (56.0-65.7)	32.8 (28.8-37.2)
Respiratory	65.1 (60.3-69.8)	37.0 (30.2-44.7)
Skin	65.2 (59.3-70.9)	34.9 (27.0-44.2)
**ALL**	**61.3 (58.8-63.7)**	**33.1 (30.3-36.0)**
Planned readmissions		
Cancers	49.7 (29.1-74.7)	68.8 (59.7-77.6)
Circulatory	19.7 (12.0-31.4)	29.5 (24.5-35.2)
Digestive	23.3 (16.9-31.6)	46.4 (40.6-52.6)
External & injuries	13.7 (11.1-16.9)	20.1 (16.9-23.7)
Genitourinary	30.0 (18.2-46.8)	43.2 (36.3-50.9)
Infections	17.0 (11.3-25.1)	31.2 (23.0-41.3)
Mental & behavioural	10.6 (8.6-13.0)	20.7 (13.6-30.9)
Musculoskeletal	19.0 (10.7-32.4)	27.7 (20.3-37.1)
Other	17.3 (13.7-21.7)	27.2 (24.2-30.4)
Respiratory	17.4 (10.9-27.2)	27.9 (21.5-35.7)
Skin	8.3 (5.3-12.8)	18.0 (11.7-27.0)
**ALL**	**16.5 (14.1-19.3)**	**30.0 (28.0-32.2)**
A&E visits		
Cancers	85.4 (74.3-93.5)	79.2 (72.1-85.5)
Circulatory	91.5 (88.1-94.2)	82.0 (77.4-86.2)
Digestive	94.9 (91.5-97.2)	83.3 (77.4-88.5)
External & injuries	93.9 (92.4-95.2)	83.1 (77.8-87.8)
Genitourinary	90.8 (79.1-97.4)	82.4 (79.1-85.5)
Infections	87.9 (77.2-95.1)	82.4 (72.7-90.2)
Mental & behavioural	95.4 (92.3-97.6)	84.9 (76.9-91.3)
Musculoskeletal	95.5 (91.5-97.9)	79.6 (69.7-88.0)
Other	94.2 (92.5-95.6)	84.3 (80.9-87.4)
Respiratory	96.7 (94.6-98.1)	88.1 (84.5-91.2)
Skin	93.7 (89.4-96.7)	83.5 (78.2-88.1)
**ALL**	**93.8 (92.7-94.8)**	**83.6 (81.2-85.8)**

Lewer D, *et al. J Epidemiol Community Health* 2021; 75:681–688. doi: 10.1136/jech-2020-215204

4 Algorithm for selecting index admission of homeless participants

Admission dates supplied by Homeless Hospital Discharge schemes did not always exactly match dates recorded in the national Hospital Episode Statistics database. In some cases homeless patients were seen by a Homeless Hospital Discharge scheme more than once. We used the following algorithm to select an index date.

Scenario	Assignment of index admission	Number of cases
One admission matches a recorded HHD	The matching admission is assigned	1507
More than one admission matches a recorded HHD	The earliest matching admission is assigned	235
No admission exactly matches	The admission closest to a recorded HHD is assigned	654
Multiple admissions are equidistant from multiple recorded HHDs	The earliest of these admissions is assigned	15
Two admissions are equidistant from one recorded HHD	One of the two admissions is assigned at random	12
No admission is within 28 days of recorded HHD	A random admission is assigned	1471

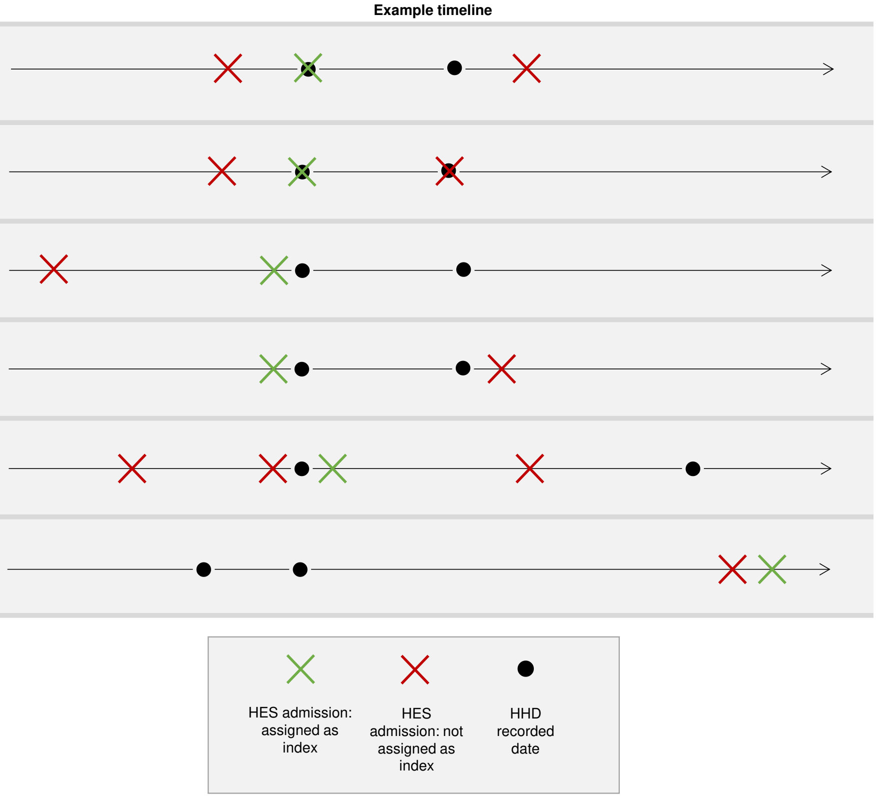

Supplement: Supplementary data [file jech-2020-215204supp001.pdf]
